# The prognostic value of 11^th^ Japanese classification and 8^th^ AJCC staging systems in Chinese patients with esophageal squamous cell carcinoma

**DOI:** 10.1186/s13019-023-02350-x

**Published:** 2023-08-23

**Authors:** Xi-qin Zhang, Chuan-wang Miao, Lan-pin Liu, Cun-liang Wang, Jia-zhen Chen, Wan-hu Li, Xu-dong Hu

**Affiliations:** 1 Shandong First Medical University, Jinan, 250000 Shandong China; 2 Department of Radiotherapy, Xingning People’s Hospital, Xingning, 514599 Guangdong China; 3 Department of Radiotherapy, Shandong Cancer Hospital and Institute, Shandong First Medical University and Shandong Academy of Medical Sciences, Jinan, 250117 Shandong China

**Keywords:** American Joint Committee on Cancer (AJCC) 8^th^ staging systems, 11th japanese classification, Overall survival, N stage, Esophageal squamous cell carcinoma (ESCC), Cancer

## Abstract

**Background:**

Two staging systems, the 8th staging system by the American Joint Committee on Cancer (AJCC) and the 11th Japanese classification by Japan Esophageal Society (JES), are currently applied in the clinic for predicting the prognosis of patients with esophageal squamous cell carcinoma (ESCC). The differences between the two staging systems have been widely researched. However, little studies focus on the differences in specific staging between the two systems. Therefore, we aimed to compare the performance of different staging in predicting overall survival (OS) of Chinese patients with ESCC.

**Methods:**

This retrospective study included 268 patients who underwent radical esophagectomy and mediastinal lymph node dissection for ESCC between January 2008 and December 2013. Patients were staged by the 8th AJCC and 11th JES staging systems. OS was estimated using the Kaplan–Meier method and compared between N stages and between stage groupings using the log-rank test. Cox proportional hazards regression analysis was performed to identify factors independently related to outcome. Further, we compared the concordance indexes (C-indexes) of the two staging systems.

**Results:**

The mean age was 61.25 ± 7.056 years, median follow-up was 44.82 months, and 5-year OS rate was 47%. The OS was well predicted by the 8th AJCC N staging (*P* < 0.001) and the 11th JES N staging (*P* < 0.001), with a c-index of 0.638 (95% CI: 0.592–0.683) for AJCC N staging and 0.627 (95% CI: 0.583–0.670) for JES N staging (*P* = 0.13). In addition, the OS was also well predicted by stage groupings of the 8th AJCC (*P* < 0.001) and the 11th JES systems (*P* < 0.001), with a c-index of 0.658 (95% CI: 0.616–0.699) for 8th AJCC stage grouping and 0.629 (95% CI: 0.589–0.668) for the11th JES stage grouping (*P* = 0.211).

**Conclusions:**

The prognostic effect of 11th JES staging system is comparable with that of AJCC 8th staging system for patients with ESCC. Therefore, both systems are applicable to clinical practice.

## Background

Esophageal cancer is one of the most common malignant tumors, and the incidence has been gradually growing worldwide [1]. China has the most significant number of cases and deaths of esophageal cancer, especially esophageal squamous cell carcinoma (ESCC). ESCC has the fourth highest mortality rate among the top 10 cancers in China [2]. Given that ESCC is life-threatening, many studies focusing on the therapies of ESCC have been done.

Cancer staging systems are critical for planning treatment and for predicting prognosis for patients with ESCC. The tumor-node-metastasis (TNM) system is the most widely used staging system developed and maintained by the American Joint Committee on Cancer (AJCC) and the International Union for Cancer Control (UICC), herein referred to as the AJCC system. It classifies cancers by the size and direct extent of the primary tumor (T), the involvement of the regional lymph nodes (N), and the presence of distant metastasis (M). The 8th edition of AJCC TNM system for esophageal cancer, based on the Worldwide Esophageal Cancer Collaboration (WECC) database, was published in 2017 [3]. In addition to the AJCC system, a novel staging system for esophageal cancer has been developed by the Japan Esophageal Society (JES). The JES system based on the JES nationwide data registry has been updated to the 11th edition in 2017 [4]. Although both the 8th AJCC and 11th JES classifications are widely applied in clinics, the two show differences in the lymph node maps, N staging, and stage grouping.

The JES system is broadly accepted in Asian countries because squamous cell cancer is the primary pathology in east Asia [5, 6]. It is also extensively used in Europe, mainly because of its detailed classification of lymph node stations [7]. By contrast, the AJCC system is used internationally as a standard scale for the staging of esophageal cancer. A retrospective study by Park et al. [8] demonstrated that the 11th JES and 8th AJCC staging systems carried similar predictive power for disease-free survival. However, Chang et al. [9] found that by comparison with the 8th AJCC staging system, the 11th JES staging system had worse performance in predicting the prognosis of patients with thoracic ESCC. There is still no consensus on which classification system is more useful in assessing patient prognosis. Therefore, in this study, we evaluated the predictive ability of these two staging systems for survival in patients with ESCC.

## Methods

### Study design and population

**Fig. 1:**
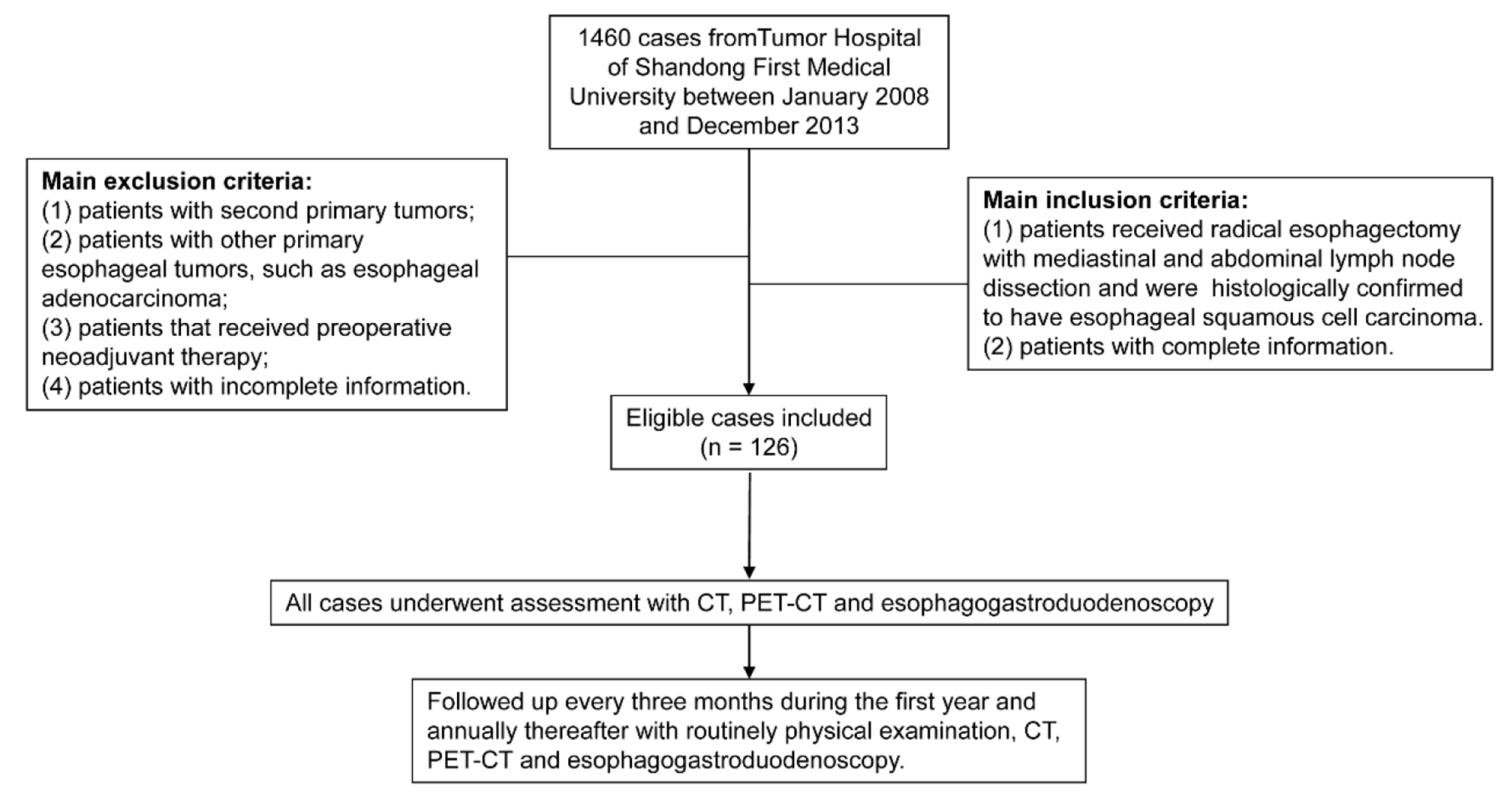
Flow chart of included patients. CT, computed tomography; PET-CT, positron emission tomography-computed tomography

A database with 1460 cases of esophageal cancer was reviewed to determine patients who underwent initial surgical treatment for esophageal cancer in the Tumor Hospital of Shandong First Medical University between January 2008 and December 2013. Clinical data of 268 cases who received radical esophagectomy (R0 resection: curative resection, the resection margin was free of cancer cells) with mediastinal and abdominal lymph node dissection and were histologically confirmed to have ESCC, were retrospectively analyzed. Exclusion of patients was based on the following criteria: (1) patients with second primary tumors; (2) patients with other primary esophageal tumors, such as esophageal adenocarcinoma; (3) patients that received preoperative neoadjuvant therapy; (4) patients with incomplete information. For data conformance, only patients with ESCC were analyzed because more than 90% of esophageal cancer is squamous cell carcinoma in east Asia. The flow chart of included patients was shown in Fig. 1.

Preoperative imaging evaluation was carried out, including chest and abdominal computed tomography (CT), positron emission tomography-computed tomography (PET-CT), and esophagogastroduodenoscopy with endoscopic ultrasound. All patients with lymph node metastasis received six cycles of adjuvant chemotherapies (paclitaxel 175 mg/m^2^ + cisplatin 75 mg/m^2^, 21 days is one cycle) after surgery. Patients staged as T2 with lymph node metastasis and T3 or T4a were treated with the same adjuvant radiotherapy (the radiotherapy dose is 70 Gy).

Patients were followed up at our outpatient clinic every three months during the first year and annually thereafter. In addition to the physical examination, chest and abdomen CT scans and PET-CT were performed during the follow-up period, and esophagogastroduodenoscopy was conducted annually following the operation. All patients included in the analysis were followed until death or December 2018. This study has been approved by the ethics committee of Tumor Hospital of Shandong First Medical University (2,021,001,008).

### Staging and lymph node map

In order to define lymph node stations accurately, lymphatic nodes were collected and classified according to the guidelines of the JES after surgery [3]. The reassessment of pathologic staging based on the 8th AJCC staging system was blinded to patient outcome, and all dissected lymph nodes were reclassified according to the 8th AJCC lymph node map of esophageal cancer [10]. Patients were assigned synchronously to the 8th AJCC staging system and 11th JES staging system according to the results of a lymph node map.

### Statistical analysis

Statistical analyses were performed using SPSS (version 22, IBM, Armonk, NY, USA). Numerical data were presented as mean with standard deviation or as median with the range. The endpoints of interest were overall survival (OS) defined as the time from initial surgery to death of any cause or to last follow-up. Survival curves were estimated using the Kaplan–Meier method and compared statistically using the log-rank test. Cox proportional hazard regression results were presented as hazard ratios (HR) with 95% confidence intervals (95% CI). Servcorp packages were responsible for the assessment and calculation of the concordance index (C-index) which was used to compare the prognostic abilities and Cox proportional hazard models of the 8th AJCC staging system and the 11th JES classification. All tests were two-sided, and *P*-value < 0.05 was considered a significant difference.

## Results

### Patient demographics and five-year survival rate

**Fig. 2:**
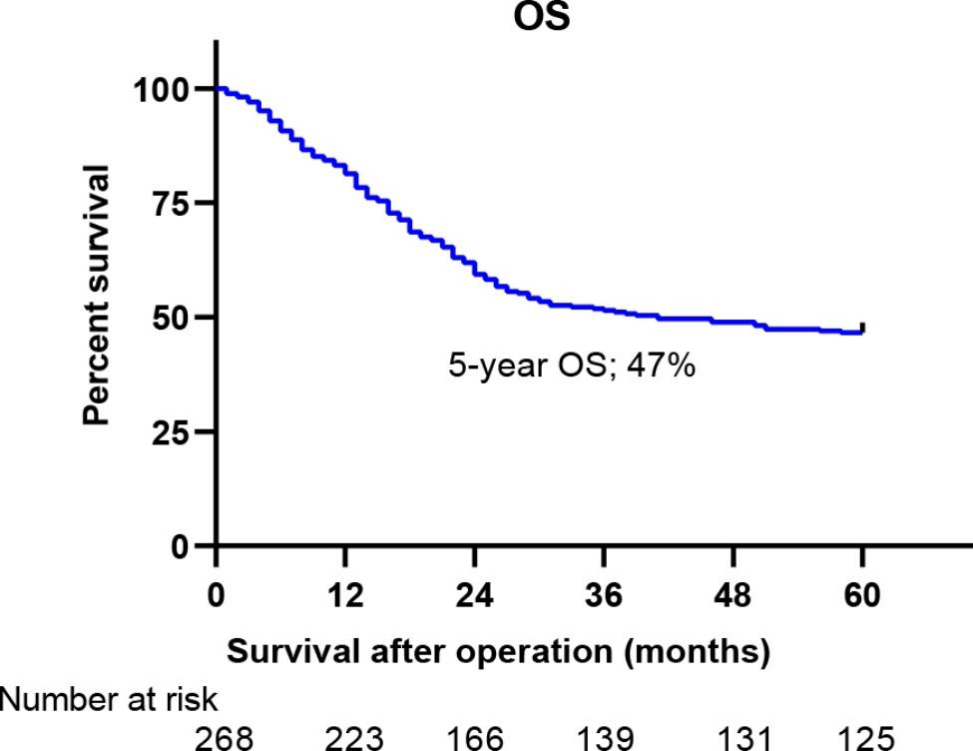
Overall survival (OS) of patients

The basic patient characteristics were obtained from 268 patients (221 males and 47 females) (Table 1). The mean age was 61.25 ± 7.056 years. The median survival time was 44.82 months, with a 5-year OS at 47% (Fig. 2). Patients were sorted by three main categories, i.e., T, N, and M staging based on the guidelines of the 8th AJCC staging system and the 11th JES staging system. As shown in *Table 2*, T staging and M staging were similar in both staging systems. However, N staging and stage grouping were different between the two staging systems.

**Table 1:** Basic characteristics of 268 patients and their tumors

Variables	Value
Age (years) Male	61.25 ± 7.056 221 (82.4%)
Female	47 (17.5%)
Location	
Upper	15 (5.5%)
Mid	176 (65.6%)
Lower	77 (28.7%)
Differentiation	
Well-differentiated	53 (19.7%)
Moderately differentiated	148 (55.2%)
Poorly differentiated	67 (25%)

**Table 2:** TNM staging based on two staging systems

Staging	AJCC 8th staging	JES 11th staging
T staging, n (%)		
0	2 (0.7%)	0 (0%)
1a	6 (2.2%)	8 (2.9%)
1b	28 (10.4%)	28 (10.4%)
2	47 (17.5%)	47 (17.5%)
3	164 (61.1%)	164 (61.1%)
4	21 (7.8%)	21 (7.8%)
N staging, n (%)		
0	140 (52.2%)	141 (52.6%)
1	73 (27.2%)	62 (23.1%)
2	36 (13.4%)	58 (21.6%)
3	19 (7.0%)	7 (2.6%)
M staging, n (%)		
0	267 (99.6%)	268 (100%)
1	1 (0.3%)	0 (0%)
Stage grouping, n (%)		
0	2 (0.7%)	8 (2.9%)
I	32 (11.9%)	21 (7.8%)
II	106 (39.5%)	119 (44.4%)
III	105 (39.1%)	120 (44.7%)
IV	23 (8.5%)	0 (0%)

AJCC, the American Joint Committee on Cancer; JES, Japan Esophageal Society

### Comparison of survival predication based on N staging between the two staging systems

**Fig. 3:**
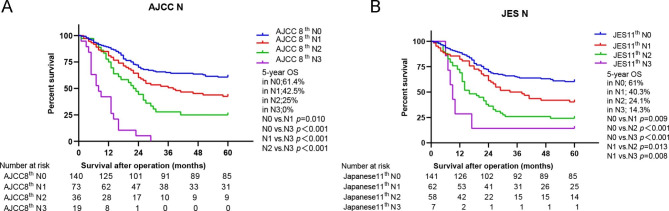
Comparison of N-stage prognosis between two staging systems. **(A)** The 5-year OS according to N stage of the AJCC 8th edition; **(B)** The 5-year OS according to N stage of the 11th JES staging. AJCC, the American Joint Committee on Cancer; JES, Japan Esophageal Society; OS, overall survival

Since N staging is the main difference between the two staging systems, survival differences were further measured according to N staging. Predication by the 8th AJCC staging system showed that the 5-year OS was 61.4% for N0, 42.5% for N1, 25% for N2, 0% for N3 (*P < 0.001*) (Fig. 3A). However, according to the 11th JES staging system, the 5-year OS was 61% for N0, 40.3% for N1, 24.1% for N2, 14.3% for N3 (*P < 0.001*) (Fig. 3B). Therefore, the prognosis of the patients with stage N3 classified by the AJCC staging system was worse than that of the patients with stage N3 classified by the JES staging system. HRs for N staging of both staging systems were calculated using univariate Cox hazards regression analysis; the higher the N stage, the higher the HRs in both of the staging systems (Table 3). Additionally, C-indexes were 0.638 (95% CI: 0.592–0.683) for the 8th AJCC N staging system and 0.627 (95% CI: 0.583–0.670) for the 11th JES N staging system. Compared to the JES N staging, the C-index of AJCC N staging was slightly higher but without significant difference (AJCC 8th vs. JES 11th, *P* = 0.13).

**Table 3:** Cox proportional model for N staging

Factors	AJCC 8th staging		JES 11th staging	
	HR (95% CI)	P	HR (95% CI)	P
N staging				
0	Reference		Reference	
1	0.109(0.063–0.188)	＜0.001	0.356(0.080–1.582)	＜0.001
2	0.183(0.104–0.322)	＜0.001	0.752(0.540–1.047)	0.091
3	0.291(0.159–0.530)	＜0.001	1.289(0.939–1.769)	0.117
Concordance index	0.638 (0.592–0.683)		0.627 (0.583–0.670)	

AJCC, the American Joint Committee on Cancer; JES, Japan Esophageal Society; HR, hazard ratio; CI, confidence interval.

### Comparison of survival predication based on staging grouping between the two staging systems

**Fig. 4:**
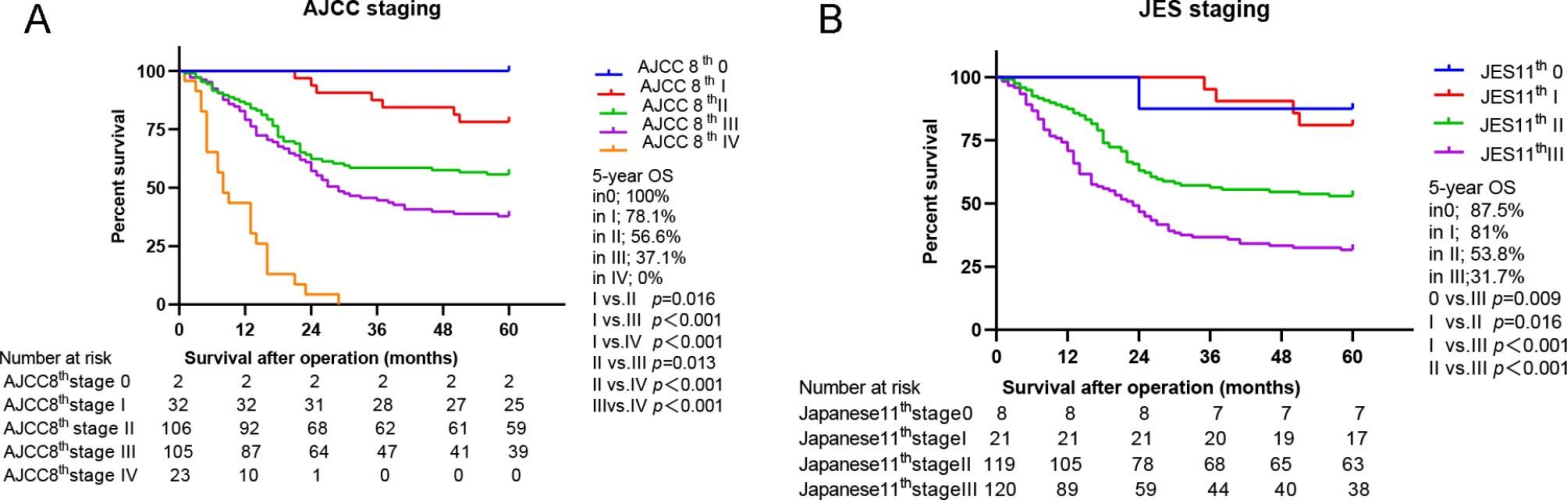
Comparison of the prognosis of the two systems according to stage grouping. **(A)** The 5-year OS according to stage grouping of the AJCC 8th staging; **(B)** The 5-year OS according to stage grouping of the 11th JES staging. AJCC, the American Joint Committee on Cancer; JES, Japan Esophageal Society; OS, overall survival

Because of the discrepancy of T, N, and M classification, the stage groupings are different between the two staging systems. Thus, survival differences were further investigated according to stage groupings. The 5-year OS predicated by the 8th AJCC staging system was 100% for stage 0, 78.1% for stage I, 56.6% for stage II, 37.1% for stage III, and 0% for stage IV (*P < 0.001*) (Fig. 4A). However, according to the 11th JES staging system, the 5-year OS was 87.5% for stage 0, 81% for stage I, 53.8% for stage II, 31.7% for stage III (*P* < 0.001) (Fig. 4B). In addition, HRs for stage groupings of both staging systems were calculated using univariate Cox hazards regression analysis; the stage groupings were directly proportional to the HRs and this was demonstrated in both of the staging systems (Table 4). C-indexes were 0.658 (95% CI: 0.616–0.699) for the 8th AJCC stage groupings and 0.629 (95% CI: 0.589–0.668) for the 11th JES stage groupings. Compared to the JES stage grouping, the C-index of the AJCC stage grouping was slightly higher but without significant difference (AJCC 8th vs. JES 11th *P* = 0.211).

**Table 4:** Cox proportional model for staging groupings

Factors	AJCC 8th staging		JES 11th staging		
	HR (95% CI)	P	HR (95% CI)	P	
Stage grouping					
0	Reference		Reference		
I	-	-	0.356(0.080–1.582)	＜0.001	
II	0.052(0.022–0.123)	＜0.001	0.528(0.217–1.282)	0.158	
III	0.133(0.079–0.225)	＜0.001	1.682(0.937–3.020)	0.081	
IV	0.215(0.131–0.354)	＜0.001	-	-	
Concordance index	0.658(0.616–0.699)		0.629(0.589–0.668)		

AJCC, the American Joint Committee on Cancer; JES, Japan Esophageal Society; HR, hazard ratio; CI, confidence interval.

## Discussion

The occurrence, development, and metastasis of esophageal cancer have certain similarities, but different pathological types have different etiology, epidemiology, and sensitivity to treatment [10–15]. The radical treatment of esophageal cancer is still surgical resection [16], but more than 60% of patients cannot receive surgical resection due to local or systemic metastasis [17]. Therefore, reasonable staging is an important way to improve the survival rate and quality of life of patients with esophageal cancer.

Both the pathological staging of surgical resection specimens and the clinical stage before surgery are hot spots of current research on esophageal cancer. The first UICC staging system and the first AJCC staging system for esophageal cancer were proposed in 1968 and in 1977, respectively [18]. However, the uniform and identical definitions and stage groupings for cancers at all anatomical sites have been introduced by AJCC and UICC since 1987 because of the close cooperation between these two organizations. The 8th AJCC/UICC staging system was proposed by WECC, which was founded in 2006 at the request by AJCC [19]. The 8th AJCC staging system was derived from a modern machine-learning analysis and a random forest analysis of data from 22,653 patients from 33 WECC institutions [20, 21]. However, Japanese data was not included in the WECC database because Japanese institutions were not affiliated to WECC [22]. The 1st edition of the Japanese classification for esophageal cancer was published by Japanese institutions in 1969, and the latest 11th edition was released in 2017 [3]. The 11th Japanese classification for esophageal cancer proposed by JES is widely accepted in Asian countries where most of the pathology is squamous cell carcinoma. However, the 8th AJCC staging system is still applied internationally as a common scale.

Each staging system has its characteristics. The most significant difference between UICC/AJCC and JES staging is the definition of N in TNM staging. The former defines N as a regional lymph node and divides N into N0-N3 according to the number of metastatic lymph nodes, while the latter divides N into N0-N4 according to the region of lymph node metastasis. In this study, a total of 268 patients were collected, including 221 men and 47 women. Their average age was 61.25 ± 7.056 years, the median survival time was 44.82 months, and the 5-year survival rate was 47%. According to the 8th AJCC staging system and the 11th JES staging system, patients were divided into three categories, namely T, N and M. The results showed that T staging and M staging were similar in the two staging systems, but differences were observed in N staging and staging grouping between the two systems. N staging of the 8th AJCC staging system based on the number of regional lymph nodes remains problematic in clinical practice. First, the connotation of supraclavicular lymph node metastasis in AJCC and JES systems is mostly different. We evaluated the prognostic significance of the supraclavicular lymph node by three-field lymph node dissection, but this evaluation method has not been adopted by western countries. Therefore, current guidelines for evaluating the prognostic significance of supraclavicular lymph nodes in ESCC are not universally applicable due to inadequate case evidence. Secondly, many metastatic lymph nodes fused are discovered during surgery, which makes the count inaccurate [23]. Thirdly, lymph node metastasis of esophageal cancer has the characteristics of biphasic and cross-cutting transfer [24, 25] and despite the rapid development of surgery, radiotherapy and radical treatment of lymph node metastases, AJCC still defines regional lymph node metastasis as distant metastasis for patients who are not in earlier T stage. Whether such a staging system can provide useful guidance for treatment and prognosis is questionable. Some Asian and western surgeons agree with their Japanese counterparts that the supraclavicular lymph nodes should be considered at least as regional lymph nodes for upper-middle thoracic esophageal cancer [24, 26–28]. The 11th JES system classifies supraclavicular nodes as group 3 nodes for lower thoracic esophageal cancer, and group 2 for upper and middle thoracic esophageal cancer. The 8th AJCC system regards patients with supraclavicular lymph node metastasis as distant metastasis requiring no surgical resection.

The selection of the staging system is related to the prediction of survival, the convenience of use, and the selection of surgical indications. Our study further studied the N stage according to the patient’s OS. According to the 8th AJCC staging system, the 5-year OS was 61.4% for N0, 42.5% for N1, 25% for N2, 0% for N3. However, according to the 11th JES staging system, the 5-year OS was 61% for N0, 40.3% for N1, 24.1% for N2, 14.3% for N3. Therefore, the prognosis of the patients with stage N3 classified by the AJCC staging system was worse than that of the patients with stage N3 classified by the JES staging system. Park et al. [7] also found that stage N3 esophageal cancer in the JES staging system had a higher survival rate than that in the AJCC staging system. In addition, univariate Cox risk regression analysis was used to calculate the HRs of N stages of the two staging systems. The results showed that the C index of AJCC N staging was slightly higher than that of JES N staging, but there was no significant difference. HRs for stage groupings of both staging systems were calculated using univariate Cox hazards regression analysis, and the results revealed that the stage groupings were directly proportional to the HRs and this was demonstrated in both of the staging systems. Compared with the JES stage grouping, the C-index of AJCC stage grouping was slightly higher, however, without any significant difference.

This study has several notable limitations. Since this is a 10-year retrospective study, there may be some confounding bias. First, we excluded neoadjuvant therapy and adenocarcinoma patients and analyzed ESCC patients only, so our results should be used cautiously for patients with esophageal adenocarcinoma. Secondly, we have not explained differences in the definition of esophagogastric junction tumors. Thirdly, we have not compared the region and degree of mediastinal and abdominal lymph node dissection. Lastly, the number of patients was relatively small. The AJCC staging system subdivided stages I, II, and III into IA, IB, IIA, IIB, IIIA, IIIB, but due to the small number of patients, we did not calculate survival differences among these subdivided stages.

## Conclusions

In conclusion, our study found that the prognostic effect of 11th JES staging system was comparable with that of 8th AJCC staging system in patients with ESCC. Therefore, both systems are applicable to clinical practice.

## Data Availability

The datasets analyzed during the current study are available from the corresponding author on reasonable request.

## Abbreviations

AJCC: American Joint Committee on Cancer
ESCC: esophageal squamous cell carcinoma
OS: overall survival
TNM: tumor-node-metastasis
UICC: International Union for Cancer Control
WECC: Worldwide Esophageal Cancer Collaboration
JES: Japan Esophageal Society
CT: computed tomography
PET-CT: positron emission tomography-computed tomography
